# Cardiorespiratory parameters and glycated hemoglobin of patients with type 2 diabetes after a rehabilitation program

**DOI:** 10.1097/MD.0000000000009321

**Published:** 2018-02-23

**Authors:** Natália da Silva Freitas Marques, Luiz Carlos de Abreu, Bárbara Vieira dos Santos, Cândido Ferreira Rodrigues Neto, José Rener Cordeiro da Silva, Karine Ketlem de Souza Braga, Kariny da Silva Uchôa, Laila Maria Silva Moraes, Leillane Cristina de Paiva Ferreira, Natanael Guimaraes Ribeiro, Savio Lima dos Santos, Tayná Almeida da Silva, Paulo Evaristo de Andrade, Rodrigo Daminello Raimundo

**Affiliations:** a Laboratory of Study Design and Scientific Writing of the Faculty of Medicine of ABC, Prince of Wales, Santo André/SP—CEP, Brazil; b Research Laboratory of UNINORTE-Baron College of Rio Branco, Alameda Hungary, Rio Branco/AC—CEP, Brazil.

**Keywords:** autonomic nervous system, exercise, glycated hemoglobin, type 2 diabetes mellitus

## Abstract

**Introduction:**

Cardiovascular autonomic dysfunction reflex of the pathophysiology of diabetes mellitus (DM) favors an increase in morbidity and mortality related to cardiovascular events, and for this reason has been one of the most studied clinical entities.

**Method:**

An experimental study of a randomized clinical trial type was therefore proposed to analyze the hemodynamic and glycemic response after the practice of a rehabilitation program in patients with type 2 diabetes mellitus (T2DM). In this clinical trial the patients will initially be submitted to an evaluation protocol that consists of assessing blood pressure, heart rate, Borg scale, respiratory rate, oxygen saturation, distance traveled through the 6-minute walk test, quality of life questionnaire, Pittsburgh sleep quality questionnaire, and still glycated hemoglobin and heart rate variability through the cardiofrequency meter. After careful evaluation of the patients, they will be submitted to a metabolic rehabilitation program composed of aerobic and resisted exercises, performed for 12 weeks, in 3 weekly meetings of 60 minutes each. With such evaluations, it will be possible to construct with evidence that it is possible to work safer metabolic rehabilitation programs in patients with T2DM or other diseases that generate cardiovascular risks, guaranteeing them an improvement in cardiorespiratory fitness, hemodynamic and glycemic variables, allowing improvement of the quality of life.

**Ethics and Dissemination:**

The protocol is approved by the host institution's ethics committee under the number 1.616.721. Results will be disseminated via peer-reviewed journal articles and conferences. This clinical trial is registered at ClinicalTrials.gov identifier: NCT3094767.

## 1 Introduction

Chronic noncommunicable diseases are the leading cause of morbidity and mortality in the world. In 2009, in Brazil, they accounted for more than 70% of all deaths, of which 31.3% were related to cardiovascular diseases (CVD). Each year it is estimated that more than 17 million people die from some CVD. Most of the risk factors for these diseases are related to living conditions and are estimated to be responsible for more than 40% of global mortality worldwide (hypertension, smoking, high blood glucose, sedentary lifestyle, overweight, and obesity).^[1]^

CVDs are the main cause of morbidity and mortality in the world.^[2,3]^ They were responsible for more than 17 million deaths in 2008, of which 3 million occurred before the age of 60, and much of it could have been avoided. The World Health Organization estimates that by 2030 almost 23.6 million people will die from cardiovascular disease.^[3,4]^

The set of cardiovascular risk factors result in the metabolic syndrome which is a complex disorder that can lead to the occurrence of CVD. Healthy living habits are strongly related to the improvement of the quality of life and positively interfere in the control of the risk factors present in this clinical condition, minimizing the chances of occurrence of CVD.^[5]^

Among the cardiovascular risk factors is diabetes mellitus (DM), characterized by a heterogeneous group of metabolic disorders resulting from defects in insulin secretion and/or action, leading to hyperglycemia.^[6]^ DM is responsible for high morbidity rates worldwide, including young individuals.^[7,8]^

In the epidemiological narrative review proposed by Petermam,^[9]^ a variation of 2% to 13% of DM in the Brazilian population in the last 3 decades was observed, being more prevalent in women, elderly, sedentary, subjects with lower educational level and those overweight or obese.

According to the International Diabetes Federation,^[8]^ an epidemic of DM is underway, with the number of diabetics worldwide being approximately 382 million, reaching 471 million in 2035.

Among the several complications from DM, neuropathies are included, and autonomic cardiac neuropathy is one of the most worrying, since it is related to the higher risk of developing cardiac diseases and mortality in this population, due to the presence of autonomic nervous system alterations.^[10–12]^

This group of patients presents an increase in morbidity and mortality related to cardiovascular events, and for this reason it has been one of the most important clinical entities.^[10–12]^ Therefore, it is understood that studies on the behavior of blood pressure (BP), and heart rate (HR), as well as the autonomic control of circulation and glycemic control in diabetic individuals, may contribute to the understanding of the pathophysiological mechanisms involved.^[13]^

The study by Lee et al^[14]^ demonstrated that regardless of the type of exercise intervention, this practice is potentially effective in improving the metabolic control of patients with type 2 diabetes mellitus (T2DM).

Therefore, to analyze the hemodynamic and glycemic behavior in relation to the real role of physical exercise in patients with T2DM, as well as all cardiovascular and metabolic diseases, will allow a more in-depth analysis of the heart rate variability (HRV), a technique used to evaluate the Performance of the autonomic nervous system, and of the metabolic markers pertinent to each disease.

The knowledge of these data can be used as a physiological parameter for the prescription of exercises, favoring the search for interventions that early improve not only the metabolic control, but also the autonomic dysfunction, guaranteeing the practice of metabolic rehabilitation programs that favor the activation of the autonomic modulation in a safety way, based on evidence, allowing improvement of their quality of life and reducing the morbidity and mortality rates related to CVD.

## 2 Method

This study protocol followed the Items of the Standard Protocol for Randomized Assays (SPIRIT). This is an experimental study of the type randomized clinical trial on the autonomic response and cardiovascular and metabolic serum markers of individuals submitted to a metabolic rehabilitation program.

### 2.1 Study population

The study population consists of diabetic patients assisted at the Health Institution called Policlínica do Tucumã, located in the municipality of Rio Branco-Acre.

### 2.2 Inclusion and exclusion criteria

Included in the research are the T2DM individuals, of both sexes, sedentary, nonsmokers, referred with medical prescription and current glycated hemoglobin result.

Patients with neurological disease, acute heart failure, type 1 diabetes, morbid obesity, atrial fibrillation, sinus arrhythmia, grade II or III atrioventricular block, smokers, and those taking beta-blockers or antiarrhythmic medication were excluded from the study.

### 2.3 Expected risks

Some risks related to the study may be: dizziness and small tiredness due to physical exercise, however in the presence of these symptoms, exercise will be interrupted until the patient is stabilized.

### 2.4 Expected benefits

It is hoped to make it possible to work cardiovascular rehabilitation programs in patients with T2DM more safely, guaranteeing them an improvement in cardiorespiratory, hemodynamic and glycemic conditioning, allowing them a better quality of life, contributing to scientific advancement.

### 2.5 Intervention

Raimundo et al,^[15]^ used a program based on 5 minutes of warm-up and 25 minutes of aerobic exercise. Such a program was adapted to increase the aerobic exercise time to 30 minutes. Therefore, individuals will be referred to a treadmill where they will begin aerobic exercise, consisting of 5 minutes of warm-up and 30 minutes of aerobic exercise, during which they will be monitored to maintain between 50% and 70% of their HR calculated individually.

After completing the aerobic training, the patients will be able to begin resistance training, which was adapted from the study by Umeda,^[16]^ which will consist of light weight exercises, comprising a load of up to 40% to 60% of that found in the test of a maximum repetition up to 3 sets of 10 repetitions increased by 10 minutes as proposed by Umeda for 20 minutes, followed by 5 minutes of deceleration.

We chose a program of aerobic and resistance exercises based on the results of the study by Church,^[17]^ where they found better results in patients with T2DM that combined aerobic activities and resistance training compared to the control group (without exercise) and also in comparison with the group that trained with aerobic or isolated resistance exercises.

The metabolic rehabilitation program consists of aerobic and anaerobic exercises performed for 12 weeks in 3 weekly meetings of 60 minutes each, totaling 36 sessions, performed in a period of 3 months.

### 2.6 Data collection

The evaluation instrument of the research will be applied before and after the practice of the proposed rehabilitation program. The instrument is based on the following collection protocol: BP, HR, Borg scale, respiratory rate, oxygen saturation, distance walked through the 6-minute walk test (6MWT), quality questionnaire (Which must be specific for each disease, and therefore the research used the diabetes quality of life-Brazil-DQOL), sleep quality questionnaire (Pittsburgh) and also the outcome variables that are glycated hemoglobin (sent by physician along with medical referral) and HRV through the cardiofrequency meter.

It is also suggested the insertion of cardiovascular and metabolic serum markers of individuals submitted to a metabolic rehabilitation program, in order to evaluate patients regarding cardiovascular risks.

**Figure 1:**
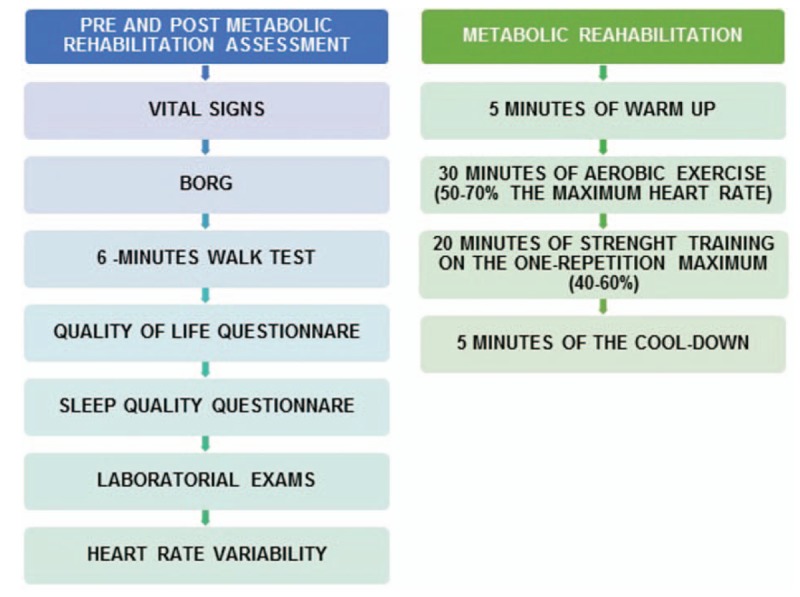
Protocol of evaluation and intervention for reduction of cardiovascular risk factors.

With this evaluation, before and after the rehabilitation program, it will be possible to observe the response of these variables (Fig. 1).

### 2.7 Instruments

The main instrument is the HRV that will be evaluated in 2 ways: at rest and in exercise. At rest, to describe the autonomic profile of diabetic and exercise patients for 2 moments (pre- and postintervention) to observe the response to the stimuli offered to the patient during the stages (warm up—5 minutes, aerobic training—30 minutes, strength training—20 minutes, cool down—5 minutes) of metabolic rehabilitation.

In the first form of collection (at rest) patients will be placed in the supine position, where they will remain for 20 minutes in an air-conditioned room (22–25°), with a capture tape attached at the time of the xiphoid process. Such a brace has 2 electrodes attached to a sealed electronic transmitter, which pick up the heart's electrical impulses to which it is transmitted to the POLAR^[18]^ heart rate monitor attached to the volunteer's wrist.

In the second form of collection (in exercise), patients will undergo a metabolic rehabilitation program, however, previously they will also position the brace at the level of the xiphoid process to record the data and then initiate the intervention. This equipment will be positioned in the patient from the beginning of the metabolic rehabilitation and will remain until the end of the recovery.

The HRV will be analyzed by means of linear methods, analyzed in the domains of time and frequency, and also by nonlinear methods and the Poincaré plot.^[19]^

The analysis of the HRV will be performed from the heart beat recorded to beat throughout the protocol, and only series with more than 95% of sinus beats will be used for analysis.^[20]^

After the error check, the trace will be selected for analysis and subjected to a digital filtering, performed in Polar Precision Performance SW software (version 4.01.029). Finally, the HRV analysis will be done using the software: Kubios.^[15]^

Another instrument used is the 6MWT, which aims to evaluate an individual's response to exercise and provides an overall analysis of the respiratory, cardiac, and metabolic systems.^[21]^ The test consisted of a walk at maximum speed and constant (without running) in a corridor of 30 m, free of human traffic and marked to each meter by yellow tape, the delimitation of the circuit was indicated by signaling cones during the test was used verbal stimuli every minute, so that the participant did not reduce the speed of the walk as: “You are walking very well, continue like this!,” “Keep up the pace,”^[22]^ and at the end, the distance traveled (DT) in meters is compared with the predicted DT that is calculated by means of the formula that indicates the distance expected for the Brazilian population: 6MWT distance (meters) = 622,461 − (1846 × age in years) + (61,503 × male = 1, female = 0).^[23]^

Still, 2 questionnaires will be applied, one that evaluates quality of life and another that evaluates the quality of sleep of the patients. The quality of life questionnaire should be specific for the metabolic alteration present, and for this study the DQOL, which is a specific instrument to measure the quality of life of patients with T2DM, will be applied. This questionnaire has 44 separate questions in 4 domains: satisfaction with 15 questions, impact with 18 questions, social/vocational concerns with 7 questions and concerns related to diabetes with 4 questions. The answers are systematized on a 5-point scale. In an intensity scale is satisfaction (1 = very satisfied, 2 = very satisfied, 3 = average satisfied, 4 = not very satisfied, 5 = not satisfied). In frequency scale are impact and concerns (1 = never, 2 = almost never, 3 = sometimes, 4 = almost always, 5 = always). The result established in these scales for better quality of life values closer to 1.^[24]^

Finally, the quality of sleep of these patients will be evaluated through the Pittsburgh Sleep Quality Index Questionnaire (PSQI) developed by Buysse.^[25]^ This instrument has 19 questions of self-report, 5 questions directed to the spouse or companion, and 5 questions that are used only for clinical practice. The 19 questions are categorized into 7 components, graded in scores of 0 (no difficulty) to 3 (severe difficulty). The 7 components are: C1 subjective sleep quality, C2 sleep latency, C3 sleep duration, C4 usual sleep efficiency, C5 sleep disturbances, C6 sleep medication used, and C7 daytime sleep dysfunction.^[26]^

The sum of the values attributed to the 7 components ranges from 0 to 21 in the total questionnaire score indicating that the higher the number the worse the quality of sleep. A total score greater than 5 indicates that the individual is experiencing major dysfunctions in at least 2 components, or moderate dysfunction in at least 3 components.^[26]^

## 3 Material

For General Cardiovascular Semiology you will need a stethoscope, sphygmomanometer, stopwatch, and thermometer.

The equipment that will be used is the Polar RS800CX heart rate receiver (RR) for the capture of the RR intervals, previously validated equipment to capture the HR beat and use of its stored data for future HRV.^[18]^ This equipment consists of 2 electrodes mounted on a sealed electronic transmitter that will be positioned in the volunteer's chest at the level of the distal third of the sternum, using an elastic band. These telemetric units will obtain the electrical impulses of the heart and will transmit such information through an electromagnetic field to the monitor that will be positioned in the wrist.^[19]^

For the 6MWT you will need a pulse meter and pulse oximeter, both handheld, stopwatch, measuring tape, signaling cones, stethoscope, sphygmomanometer, symptom perception scale printed on plasticized paperboard, suitable size and allowing easy reading and chair along if the patient needs rest.

After conducting the evaluation, equipment will be needed for the implementation of the metabolic rehabilitation program. The necessary equipments are: treadmill, cardiofrequencimeter (to monitor the training), dumbbells and shiners of the most diverse loads to respect the result of the test of a maximum repetition of each individual.

## 4 Measures

### 4.1 Primary outcomes

Recruitment began in September 2016, and is underway. A total of 9 patients were evaluated and submitted to the intervention protocol by the end of June 2017.

### 4.2 Secondary outcomes

The protocol may provide subsidy for implantation of metabolic rehabilitation programs in patients with other CVD such as dyslipidemia, hypertension among others.

### 4.3 Data analysis

Data will be expressed as mean and standard deviation. The SPSS software version 24.0 will be used for statistics. The descriptive data will be measured by measures of central tendency and dispersion. The Shapiro–Wilk test will be used to verify the normality of the data. To compare the cardiorespiratory parameters, we will use the Friedmann test or the ANOVA test of repeated measures according to the normality of the data. The level of significance will be *P* < .05. All values will be compared with the rest condition.

## 5 Ethics and dissemination

The study protocol was submitted and approved by the Research Ethics Committee of the Hospital das Clínicas do Acre through Opinion No. 1,616,721. Only participants who agree to provide written informed consent will be entered into the survey.

This registry of clinical trials was titled “Cardiorespiratory Parameters and Glycated Hemoglobin of Patients With Type 2 Diabetes After a Rehabilitation Program,” on March 28, 2017 in “ClinicalTrials.Gov” (NCT03094767).

## 6 Discussion

The presence of cardiovascular risk factors as well as the presence of CVD contribute to an increase in the number of deaths, hospitalizations, and disabilities in the adult population. In view of this, primary care assistance with health promotion can be considered as the response to the modification of modifiable factors that influence the installation of risks and CVD, improving living conditions and creating favorable environments for health.^[27]^

The health promotion strategy represents an assertive alternative for addressing these risk factors.^[1]^ However, the multidisciplinary and transdisciplinary approach strengthens the actions in health, since the collaborative posture reinforces the purposes of prevention, control, and treatment of diseases. However, the inclusion of a protocol that includes a detailed evaluation followed by the implementation of metabolic rehabilitation will contribute to the construction of patient-centered care and its needs in order to improve people's health conditions.^[4,28]^

## References

[1] Audi CA Santiago SM Andrade MD Fatores de risco para doenças cardiovasculares em servidores de instituição prisional: estudo transversal. Epidemiol Serv Saúde 2016;25:301–10. 27869948 10.5123/S1679-49742016000200009

[2] Monteiro Junior FC Mandarino NR Salgado JVL Deficiência de vitamina D: um novo fator de risco cardiovascular?/vitamin D deficiency: a new cardiovascular risk factor? Rev Bras Cardiol (Impr) 2014;27:356–65.

[3] World Health Organization. Global Status Report on Noncommunicable Diseases 2010 [Internet]. Geneva: World Health Organization; 2011.

[4] Radovanovic CAT Santos LA Carvalho MDB Hipertensão arterial e outros fatores de risco associados às doenças cardiovasculares em adultos. Rev Latino-Am Enfermagem 2014;22:547–53.

[5] Soares TS Piovesan CH Gustavo Ada S Alimentary habits, physical activity, and Framingham global risk score in metabolic syndrome. Arq Bras Cardiol 2014;102:374–82. 24652053 10.5935/abc.20140029 PMC4028945

[6] Diabetes Brazilian Society (SBD). Guidelines of the Brazilian Society of Diabetes. Sao Paulo: Jose Egidio Paulo de Oliveira, Sergio Vencio; 2015.

[7] Costa MC Brito LL Lessa L Práticas alimentares associadas ao baixo risco cardiometabólico em mulheres obesas assistidas em ambulatórios de referência do Sistema Único de Saúde: estudo de caso-controle. Epidemiol Serv Saúde 2014;23:67–78.

[8] International Diabetes Federation. IDF Diabetes Atlas. Belgium: IDF. 2013.

[9] Petermam XB Machado IS Pimentel BN Epidemiologia e cuidado à diabetes mellitus praticado na Atenção Primária à Saúde: uma revisão narrativa. Saúde (Santa Maria), Santa Maria 2015;41:49–56.

[10] Vinik AI Maser RE Mitchell BD Diabetic autonomic neuropathy. Diabetes Care 2003;26:1553–79. 12716821 10.2337/diacare.26.5.1553

[11] Duncan JG Mitochondrial dysfunction in diabetic cardiomyopathy. Biochim Biophys Acta 2011;1813:1351–9. 21256163 10.1016/j.bbamcr.2011.01.014 PMC3149859

[12] Capote, AE. Efeitos do diabetes mellitus experimental sobre a evolução temporal da variabilidade da frequência cardíaca. Dissertação de Mestrado em Fisiologia—Universidade Federal do Paraná, Coimbra, 2009.

[13] Angelis KD Schaan BA Rodrigues B Disfunção autonômica cardiovascular no diabetes mellitus experimental. Arq Bras Endocrinol Metab 2007;51:185–94. 10.1590/s0004-27302007000200007 17505625

[14] Lee SF Pei D Chi MJ An investigation and comparison of the effectiveness of different exercise programmes in improving glucose metabolism and pancreatic b cell function of type 2 diabetes patients. Int J Clin Pract 2015;69:1159–70. 26119968 10.1111/ijcp.12679

[15] Raimundo RD de Abreu LC Adami F Heart rate variability in stroke patients submitted to an acute bout of aerobic exercise. Transl Stroke Res 2013;4:488–99. 24323375 10.1007/s12975-013-0263-4

[16] Umeda IIK Manual de Fisioterapia na Reabilitação Cardiovascular. 2nd ed. Barueri: Manole; 2014.

[17] Church TS Blair SN Cocreham S Effects of aerobic and resistance training on hemoglobin A1c levels in patients with type 2 diabetes: a randomized controlled trial. JAMA 2010;304:2253–62. 21098771 10.1001/jama.2010.1710 PMC3174102

[18] Andrade PE Amaral JT Paiva LS Reduction of heart rate variability in hypertensive elderly. Blood Pressure 2017;3:1–9. 10.1080/08037051.2017.1354285 28738697

[19] Vanderlei LCM Pastre CM Hoshi RA Noções básicas de variabilidade da frequência cardíaca e sua aplicabilidade clínica. Rev Bras Cir Cardiovasc 2009;24:205–17. 19768301 10.1590/s0102-76382009000200018

[20] Godoy MF Takakura IT Correa PR Relevância da análise do comportamento dinâmico não-linear (Teoria do Caos) como elemento prognóstico de morbidade e mortalidade em pacientes submetidos à cirurgia de revascularização miocárdica. Arq Ciênc Saúde 2005;12:167–71.

[21] Morales-Blanhir JE Vidal CDP Romero MJR Teste de caminhada de seis minutos: uma ferramenta valiosa na avaliação do comprometimento pulmonar. J Bras Pneumol 2011;37:110–7.21390439

[22] Rondelli RR Oliveira AN Corso SD Uma atualização e proposta de padronização do teste de caminhada dos seis minutos. Fisioter Mov 2009;22:249–59.

[23] Iwama AM Andrade GN Shima P The six-minute walk test and body weight-walk distance product in healthy Brazilian subjects. Braz J Med Biol Res 2009;42:1080–5. 19802464 10.1590/s0100-879x2009005000032

[24] Correr CJ Pontarolo R Melchiors AC Tradução para o Português e Validação do Instrumento Diabetes Quality of Life Measure (DQOL-Brasil). Arq Bras Endrocrinol Metab 2008;52:515–22. 10.1590/s0004-27302008000300012 18506277

[25] Buysse DJ Reynolds CF Monk TH The Pittsburgh sleep quality index: a new instrument for psychiatric practice and research. Psychiatry Res 1989;28:193–213. 2748771 10.1016/0165-1781(89)90047-4

[26] Araújo PAB Qualidade do sono de participantes de programa de reabilitação cardiopulmonar e metabólica. Cinergis 2015;16:102–6.

[27] Ferreira CCC Peixoto MRG Barbosa MA Prevalência de fatores de risco cardiovascular em idosos usuários do sistema único de saúde de goiânia. Arq Bras Cardiol 2010;95:621–8.21109910

[28] Gomes CM Capellari C Pereira DSG Estresse e risco cardiovascular: intervenção multiprofissional de educação em saúde. Rev Bras Enferm 2016;69:351–9. 27280572 10.1590/0034-7167.2016690219i

